# Esophageal Stricture in a child with Recurrent Oropharyngeal Candidiasis With *Pseudomonas aeruginosa* Esophagitis

**DOI:** 10.1097/PG9.0000000000000013

**Published:** 2020-09-22

**Authors:** Naina Chakravarty, Vishrutha Poojari, Ira Shah

**Affiliations:** From the Department of Pediatric Gastroenterology and Hepatology, BJ Wadia Hospital for Children, Mumbai, India

Esophageal stricture, defined as an abnormal narrowing of the esophageal lumen generally due to an intraluminal pathology, is usually seen in pediatric age group due to corrosive substance ingestion, eosinophilic esophagitis, radiation injury, or infectious esophagitis (1). The primary infectious causes of esophagitis are fungal and viral. There are also case reports of esophagitis caused by bacteria or parasites, but these are quite rare. Esophageal infections are usually only seen in immunosuppressed children (1). Infectious esophagitis with chronic inflammation may uncommonly progress to fibrosis and stricture as it induces sloughing of the esophageal mucosa (2). There have been very few patients with esophageal stricture due to infectious esophagitis reported in adults and even less in pediatrics, mostly all developed during treatment for acute lymphoblastic leukaemia (3). We present a 3½-year-old boy with recurrent oropharyngeal candidiasis with *Pseudomonas aeruginosa* esophagitis who developed esophageal stricture and had an excellent response to endoscopic controlled radial expansion balloon dilatation.

## CASE REPORT

**FIGURE 1.**
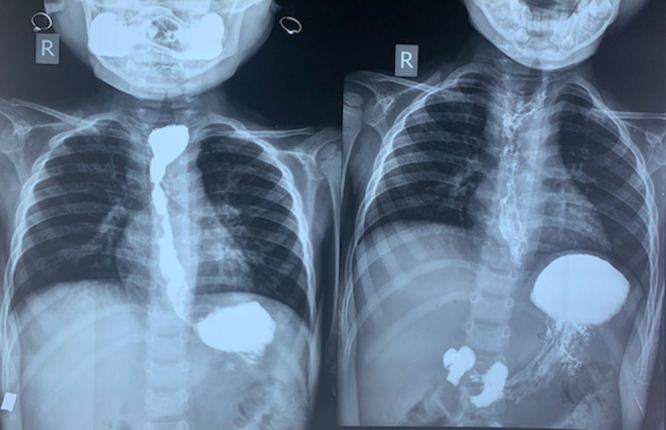
Barium swallow

**FIGURE 2.**
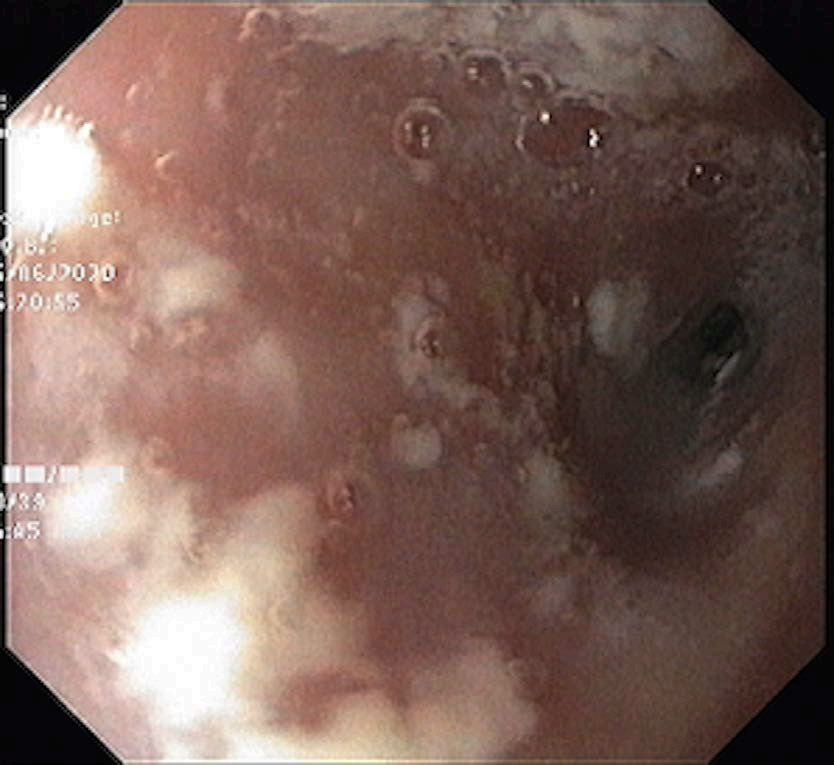
Esophagus with white patches and narrow stricture.

**FIGURE 3.**
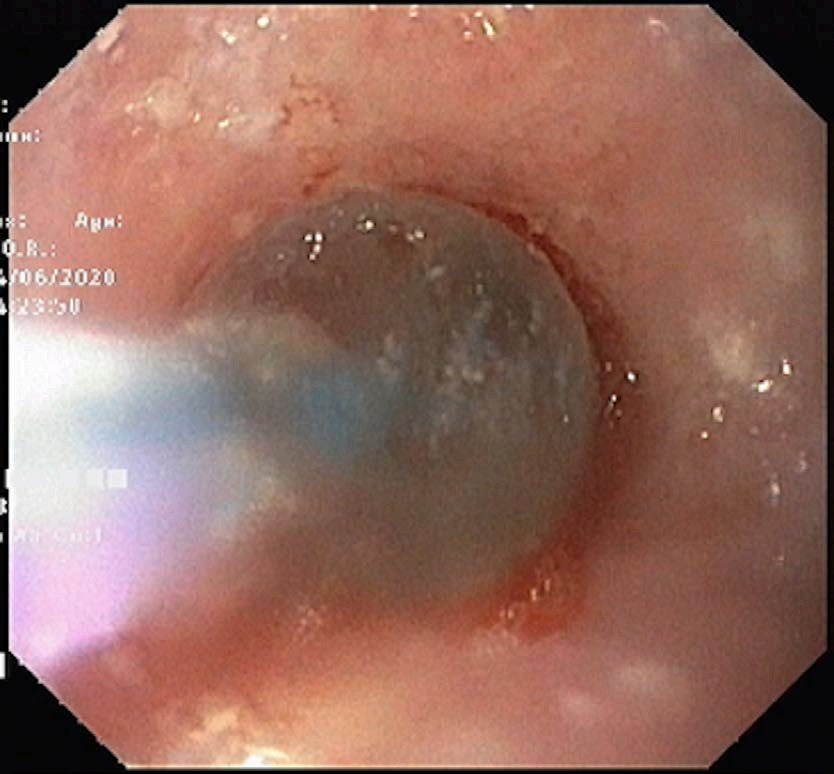
CRE balloon dilatation. CRE = controlled radial expansion.

A 3½-year-old boy presented to us in May 2020 with dysphagia to solids since he was 1 year old. The patient was diagnosed as having oral candidiasis at 3 months of age, which was treated by topical clotrimazole at the time. He continued to have recurrent episodes of oral candidiasis over the next 3 years, which were treated with oral fluconazole initially and then with oral voriconazole. The patient was also admitted twice with lower respiratory tract infections requiring oxygen support and intravenous (IV) antibiotics and recurrent episodes of otitis media. The child accepted semisolid foods when he was weaned at 6 months of age but when solids were tried at 1 year, he had multiple episodes of spitting up, which were clinically suspected as gastroesophageal reflux disease (GERD) at the time. However, despite treatment for GERD with lanzoprazole and domperidone, there was no improvement in dysphagia and the patient also had poor weight gain. Immunodeficiency workup in form of serum immunoglobulins (IgA, IgG, IgE, IgM), lymphocyte subsets, and nitro blue tetrazolium slide tests were normal. Human immunodeficiency virus ELISA was negative. On presentation to us, weight was 8.3 kg (<third percentile as per Indian Academy of Pediatrics growth charts) and height was 86 cm (<third percentile as per Indian Academy of Pediatrics growth charts). He had oral thrush and right ear discharge. Other systems were normal. Investigations showed hemoglobin 10.1 gm/dL, total leucocyte count 8600 cells/cumm, platelets 400,000 cells/cumm. Ultrasonography abdomen was normal. Barium swallow showed presence of long-length stricture in the mid esophagus (Fig. 1). Upper gastrointestinal scopy was performed by Olympus GIF/XP 170 N pediatric scope and showed white patches in the oropharynx and the esophagus (Fig. 2). A tight long-length 4 cm stricture was seen 11 cm from the incisors. Esophageal biopsy was sent for culture; however, it showed no fungal growth but growth of *P. aeruginosa*. Pus culture from ear discharge, grew of *P. aeruginosa.* He was treated with IV ceftazidime and ciprofloxacin along with liposomal amphotericin B (3 mg/kg/d) for 14 days. As there was persistence of white patches in the oropharynx and the esophagus despite 14 days of IV amphotericin B, antifungals were changed to IV caspofungin (loading dose 70 mg/m^2^ and then 50 mg/m^2^/d) and oral voriconazole (9 mg/kg/dose twice a day) for 14 days to which he responded. Endoscopic sequential esophageal stricture dilatation was started after 2 weeks of IV antibiotics, and the stricture was dilated upto 15 mm using controlled radial expansion balloons over a period of 1 month (Fig. 3). Post dilatation, the child is able to eat solids and weight has increased by 1 kg. Oral voriconazole (6 mg/kg/dose twice a day) and cotrimoxazole (5 mg/kg/d) as prophylaxis have been continued. He was advised regular follow up.

## DISCUSSION

Although, a rare complication, esophageal stricture should be considered in an immunosuppresed patient who continues to have dysphagia despite appropriate treatment. Esophageal stricture following fungal esophagitis has been reported in a 3-year-old girl with leukaemia and dysphagia (4). Lee et al in 2016 reported the first case of esophageal stricture secondary to candidiasis in a glycogen storage disease 1b child (5). Our patient had recurrent oropharyngeal candidiasis for more than 3 years and presented with dysphagia and stricture. Zhang et al reported esophageal stricture secondary to actinomyces in a 45-year-old female (6). Tuberculosis is a rare cause of esophageal stricture. Shin et al (7) reported group B Streptococcus ulcerative esophagitis that led to stricture formation. Similarly our patient grew *P. aeruginosa* on culture from the esophageal biopsy.

The diagnosis of esophageal stricture should be considered in all patients presenting with dysphagia and having history of frequent oropharyngeal infections. After a thorough medical history and bedside evaluation, if there is suspicion of esophageal stricture, the next best investigation would be an esophagogastroduodenoscopy or contrast-enhanced esophagogram. Endoscopy is preferred since it can provide overall information on esophageal anatomy and establish not just the diagnosis of a stricture but also allow for biopsy of the mucosa and offers an opportunity for therapeutic dilation of the stricture when indicated (1).

Till date the first line management for benign esophageal strictures remains dilatation via a balloon or bougie with or without steroid injection (1). However, Kalla et al (8) reported a case of candidal esophagitis leading to stricture, which resolved with a prolonged course of fluconazole therapy without requiring endoscopic dilatation. In our patient, probably the stricture was long standing as the patient had dysphagia for 2 years and thus intervention was required.

## CONCLUSION

Esophageal stricture should be suspected in patients with recurrent oropharyngeal infections and dysphagia to avoid misdiagnosis of GERD. Although fungal infections are common causes of esophagitis, bacterial causes of esophagitis should also be considered.
